# Low frequency oscillations assessed by diffuse speckle contrast analysis for foot angiosome concept

**DOI:** 10.1038/s41598-020-73604-0

**Published:** 2020-10-13

**Authors:** Chaebeom Yeo, Hanbeen Jung, Kijoon Lee, Cheol Song

**Affiliations:** 1 Department of Robotics Engineering, Daegu Gyeongbuk Institute of Science and Technology, Daegu, 42988 Republic of Korea; 2 School of Undergraduate Studies, Daegu Gyeongbuk Institute of Science and Technology, Daegu, 42988 Republic of Korea

**Keywords:** Optical spectroscopy, Biomedical engineering, Diabetes

## Abstract

An angiosome refers to a 3D tissue volume that is vascularized by a single artery and is a relatively new concept that is useful in vascular surgery; however, the direct relationship between arterial blood flow and micro-perfusion is still controversial. Here, we propose a diffuse speckle contrast analysis (DSCA), which is an emerging tissue perfusion monitoring modality, to investigate the correlations among low frequency oscillations (LFOs) measured from different areas on the feet of healthy subjects. We obtained reproducible results from the correlation analyses of LFOs, and their physiological implications were discussed. In order to confirm the changes in the frequency oscillations, we analyzed and compared the power spectral density changes due to heart rate variability in the electrocardiographic signal during reactive hyperemia and head-up tilt protocols.

## Introduction

Critical limb ischemia (CLI) is one of the complications of diabetes, although it is not the unique complication, and the population of people who are affected by this condition is gradually increasing along with the incidence of diabetes^1,2^. Severe diabetes mellitus can lead to diabetic foot ulcers and may necessitate lower extremity amputations^3^. In the context of treating the feet of patients with CLI, the angiosome concept has recently gained popularity in the field of vascular surgery. The angiosome concept was first devised by Tayler and Palmer in 1987 for vascular surgical therapy^4^. According to this concept, a single source artery feeds a specific three-dimensional volume of tissue. As a representative example in the leg, the femoral artery in the thigh extends to three different angiosomes related to the anterior tibial artery (ATA), posterior tibial artery (PTA), and peroneal artery (PA). This concept was further extended by Attinger et al.^5^ for peripheral revascularization in patients with CLI. They reported six angiosomes in the foot and lower leg, where the PTA particularly branches into three angiosome spaces, namely the medial plantar branch (MPB), lateral plantar branch (LPB), and calcaneal branch (CB) of the PA, which are responsible for circulation in the sole and heel of the foot. From this foot angiosome concept, the ischemic artery that is responsible for foot ulcers can be estimated, and strategies of vascular surgery can be established^4–9^. However, it is still debatable whether the angiosome concept is applicable to the feet of patients with CLI^10–12^ because new collateral vessels may replace the original arterial supply to skin areas after vessel occlusion.

In the walls of blood vessels, vasomotion oscillations (i.e., spontaneous systemic changes) occur with various frequencies, including in the range of heart beats (~ 1 Hz), high frequencies (HFs; range: 0.15–0.4 Hz) that are produced by respiration, low frequencies (LFs; range: 0.04–0.15 Hz) that are closely related to Mayer waves, and very low frequencies (VLFs; range: ≤ 0.04 Hz) that are much less studied^13^. Amplitude changes in VLFs and LFs, which are obtained by functional magnetic resonance imaging^14,15^ and near-infrared spectroscopy^16–18^, following specific tasks or stimulations have recently garnered interest in hemodynamic and neuronal connectivity studies. Some studies^17,19^ have termed the combined variations in VLF and LF bands as low frequency oscillations (LFOs; range: 0.01(or 0.04)–0.15 Hz.

Diffuse speckle contrast analysis (DSCA) is an optical methodology that is capable of estimating tissue perfusion changes using correlations in speckle intensities^20^. This system has been recently validated for a variety of biomedical samples^20–25^; it has advantages such as fast signal processing, simple experimental setup and signal analysis, and relatively deep tissue perfusion measurements compared to other blood flow instrumentations. In this study, we employed the DSCA with four-channel optical probes to concurrently obtain LFOs ranging from VLFs to LFs from four sites on the foot, based on the foot angiosome concept. The measurement for LFO in human arm using DSCA has been previously reported by Bi et al.^20^.

As the first objective of this study, we examined the correlations among the LFOs from the four different areas of the foot (i.e., two channels of the optical probes were located in the LPB angiosome and the remaining two channels were located in the MPB angiosome) in healthy subjects, in order to validate whether the foot angiosome concept is appropriate in direct relationships between arterial blood flow and microcirculation. Our hypothesis is that if the angiosome concept is true, the correlations of the LFOs within each of the angiosomes will be high as vasculature from an angiosome share the same artery. The second objective of this study was to evaluate the relevance of the LFOs measured by DSCA; for this purpose, we analyzed the power spectral density (PSD) changes in the frequency oscillations following reactive hyperemia and 90° head-up tilt protocols by comparing with the PSD changes of VLFs, LFs, and HFs from heart rate variability (HRV) data obtained by electrocardiography (ECG).

## Results

### Cross-correlation based on foot angiosome concept

#### DSCA representative results from one subject

**Figure 1:**
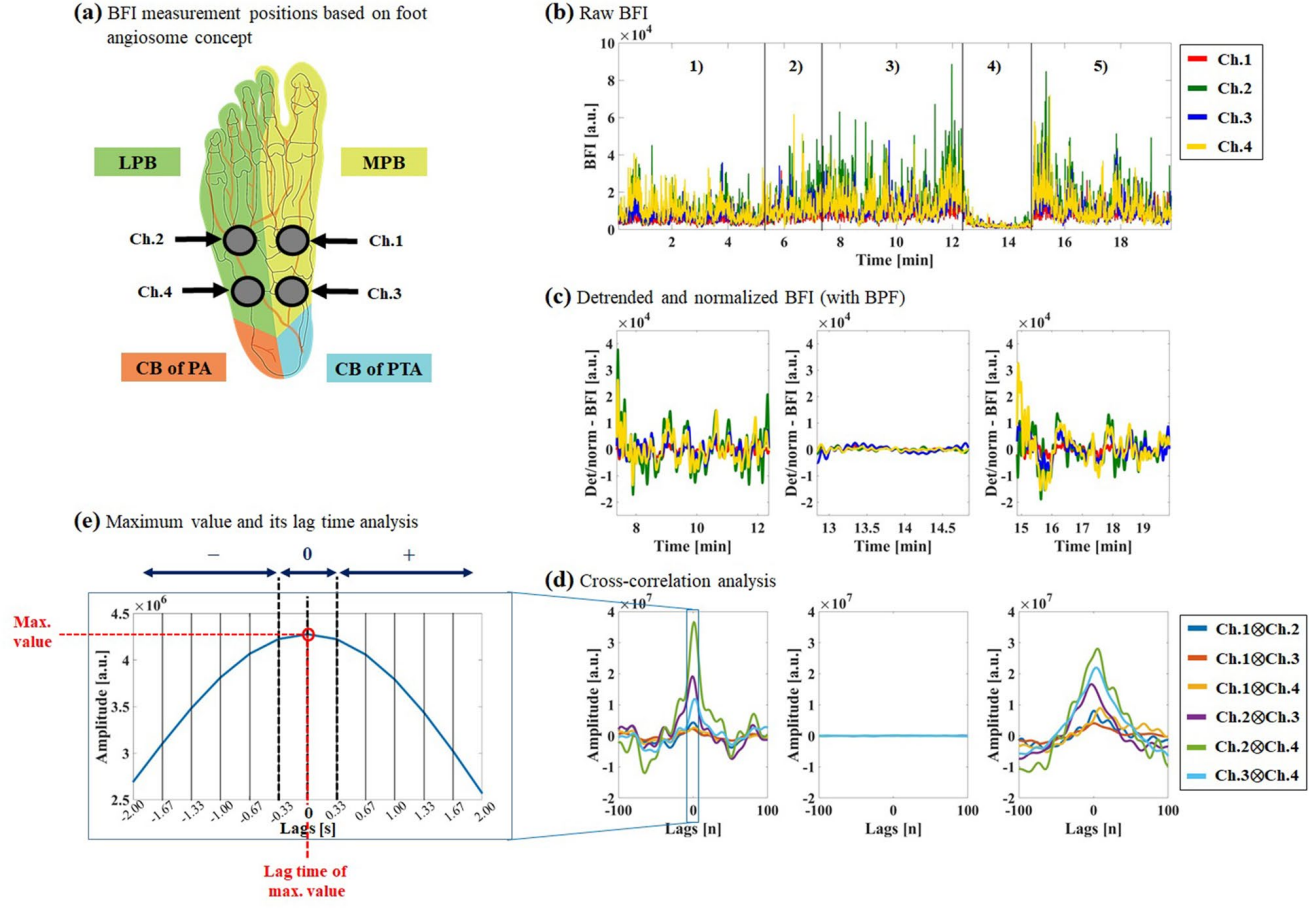
DSCA representative results of cross-correlation analysis in one subject according to baseline, occlusion, and release periods at supine posture. (**a**) Measurement positions of blood flow index (BFI) based on foot angiosome with four-channel DSCA probes on subject’s sole. (**b**) Raw BFI data. (1) Baseline period in tilt posture. (2) Posture change period. (3) Baseline period in supine posture. (4) Cuff-occlusion period in supine posture. (5) Release period in supine posture. (**c**) Detrended, normalized (processed), and filtered BFI with band pass filter (BPF) based on sectioned BFIs (Left: Baseline period in supine posture; Center: Cuff-occlusion period in supine posture; Right: Release period in supine posture). (**d**) Cross correlation analyses among four channels based on (**c**). (**e**) Maximum value and its lag time analysis in Ch.1 $\otimes$ Ch.2 relationship.

In order to analyze the cross correlations among the four different LFOs obtained from the four channels of the DSCA system, we first examined the time-series data of the blood flow index (BFI) in all the optical channel probes. Figure 1 shows the representative results measured by DSCA from a single subject’s foot. Figure 1(a) shows the four channels (from Ch.1 to Ch.4) of the DSCA that were used to obtain data from the sole, based on the foot angiosome concept introduced by Attinger et al.^5^. As shown in Fig. 1(b), we were able to observe tissue perfusion changes in all the optical channels by changing the body posture (from 90° head-up tilt to supine) and by controlling the blood pressure at the subject’s thigh. Although all the BFIs for both postures seemed to have similar tissue perfusion trends during the baseline period, these BFIs showed dramatic decreases and increases according to cuff-occlusion and release of occlusion, corresponding to autoregulation of blood flow, from which we determined that the DSCA measurement was sensitive to hemodynamic changes in the human foot. Figure 1(c) shows the sectioned timeline BFI data for the baseline (supine), occlusion, and release periods after detrending, normalization, and band pass filter (BPF; pass band frequencies: 0.01–0.15 Hz). Since all BFIs on the four channels fluctuated in a similar manner (i.e., showed LFOs), it was hard to recognize the highly correlated pair intuitively. Figure 1(d) shows the six cross correlations from the four channels for the baseline, occlusion, and release periods, respectively. With the exception of the cross-correlation analysis for the occlusion period, the peaks from all six pairs were distributed around zero lag. In the occlusion period, since the amplitudes of the BFI and LFO decreased owing to the decrease of blood flow supply and autonomic nervous activity, the meaningful relationships disappeared, and we were unable to perform quantitative analyses of the cross correlations. Figure 1(e) illustrates how to obtain the maximum value and its lag time from the cross-correlation graph.

**Table 1:** Maximum values, its rank, and lag times from six cross-correlation analysis according to baseline and release periods at supine posture in one subject’s foot.

Periods	Contents	Ch.1 $\otimes$ Ch.2	Ch.1 $\otimes$ Ch.3	Ch.1 $\otimes$ Ch.4	Ch.2 $\otimes$ Ch.3	Ch.2 $\otimes$ Ch.4	Ch.3 $\otimes$ Ch.4
Baseline	Max. value [a.u.]	$4.28 \times 10^{6}$	$2.20 \times 10^{6}$	$2.63 \times 10^{6}$	$19.14 \times 10^{6}$	$36.61 \times 10^{6}$	$11.88 \times 10^{6}$
	Rank	4	6	5	2	1	3
	Lag time [s]	0	− 0.33	0.67	−0.33	0.33	0.67
Release	Max. value [a.u.]	$8.03 \times 10^{6}$	$4.03 \times 10^{6}$	$8.96 \times 10^{6}$	$16.62{ } \times 10^{6}$	$28.05 \times 10^{6}$	$21.94 \times 10^{6}$
	Rank	5	6	4	3	1	2
	Lag time [s]	0.33	0	3	−1	1.67	1.33

As shown in Table 1, we analyzed the maximum values for each of the six cross correlations for the baseline and release periods. Then, the ranks and lag times for the maximum values were estimated; from the high ranks among the six relationship pairs, we noted that three pairs (Ch.2 $\otimes$ Ch.3, Ch.2 $\otimes$ Ch.4, and Ch.3 $\otimes$ Ch.4) were highly correlated regardless of the impact of the reactive hyperemia protocol. Compared to the previous hypothesis that Ch.1 $\otimes$ Ch.3 and Ch.2 $\otimes$ Ch.4 relationships will show relatively high cross correlations; the obtained result was not fully compatible with the foot angiosome concept.

From the cross-correlation analysis in this study, it is possible to estimate the similarities between the tissue perfusion data acquired from the optical channels by measuring how large their values are. Moreover, the time latencies (i.e., lag times) for hemodynamic responses can be estimated via the lead–lag time analysis. Briefly, from the locations of the maximum values with respect to zero lag, the leading or lagging of a specific channel pair can be determined. The negative sign in the lag time indicates that the second channel of the pair is faster, and vice versa. The meaning of zero in lag time analysis is that there is no lag latency between the two channels in the pair within our system resolution. Since the data sampling speed of the DSCA is 3 Hz, each data point of the lag number indicates a time duration of 0.33 (= 1/3) s, as shown in Fig. 1(e).

#### DSCA results of statistical analysis in all subjects

For the cross-correlation data from all the subjects (N = 10), we performed statistical analyses with the averages and standard deviations of the ranks for the maximum values from among the six cross correlations at two different periods and postures, as summarized in Table 2. From the rank analysis, we found that there were no significant differences compared to the previous representative results (Table 1), thereby showing high ranks for the average maximum values for three relationships (Ch.2 $\otimes$ Ch.3, Ch.2 $\otimes$ Ch.4, and Ch.3 $\otimes$ Ch.4). Furthermore, depending on baseline/release periods and supine/tilt postures, the ranks of the cross correlations were not significantly different from each other.

**Table 2:** Average and standard deviation of the rank for the maximum value from six cross-correlation analyses at baseline and release periods in all subjects, according to supine and 90° head-up tilt posture.

Posture	Periods	Ch.1 $\otimes$ Ch.2	Ch.1 $\otimes$ Ch.3	Ch.1 $\otimes$ Ch.4	Ch.2 $\otimes$ Ch.3	Ch.2 $\otimes$ Ch.4	Ch.3 $\otimes$ Ch.4
Supine	Baseline	4.65 ± 0.86	4.82 ± 0.70	4.62 ± 0.75	2.32 ± 0.89	2.15 ± 0.93	2.45 ± 0.71
	Release	4.21 ± 1.11	4.70 ± 0.71	5.03 ± 0.56	2.13 ± 0.70	1.93 ± 0.40	2.98 ± 0.94
Tilt	Baseline	4.43 ± 0.59	5.10 ± 0.63	4.97 ± 0.64	2.10 ± 0.67	1.97 ± 0.60	2.53 ± 0.61
	Release	3.72 ± 1.10	4.92 ± 0.58	5.13 ± 0.59	1.98 ± 0.69	2.07 ± 0.64	3.18 ± 0.75

Once the statistical analysis for the lag time was performed with the average and standard deviation calculations for all subjects, the deviations were observed to be too high to obtain meaningful results because the lag time for each subject was different. Instead, as shown in Table 3, we counted the number of occurrences of each sign (+ : positive, − : negative, 0: zero, as shown in the top part of Fig. 1(e)) in the sixty outcomes for the baseline and release periods in all subjects (i.e., 60 = 2 (two different periods) × 10 (ten subjects) × 3 (repeat measurement 3 times). Then, we calculated the percentages for each sign depending on the supine and 90° head-up tilt postures. From this analysis, we derived two reproducible results related to hemodynamic responses among all the channel pairs, based on the angiosome concept. The first result was that Ch.1 and Ch.3 in the MPB angiosome space are faster than Ch.2 and Ch.4 in the LPB angiosome, regardless of supine/tilt posture. In the relationships linked with channel 3, except for the Ch.1 $\otimes$ Ch.3 pair, the highest percentages for the lag time signs in the Ch.2 $\otimes$ Ch.3 pair were all negative at both supine and tilt postures, whilst they were all positive for the Ch.3 $\otimes$ Ch.4 pair, showing the highest percentages were above 58.33%. In other words, the blood supply recorded at channel 3 is faster than those at channels 2 and 4. For the relationships linked with channel 1, except for Ch.1 $\otimes$ Ch.3, the highest percentages for both Ch.1 $\otimes$ Ch.2 and Ch.1 $\otimes$ Ch.4 relationships were positive at both supine and tilt postures, suggesting that the blood in channel 1 flowed earlier than those in channels 2 and 4. For the second result, in the relationships between two channels in the same angiosome (i.e., Ch.1 $\otimes$ Ch.3 and Ch.2 $\otimes$ Ch.4), the sign dominance for positive/zero/negative was unclear depending on the supine and tilt postures, and the highest percentages showed below 51.67%. The time latency analysis of the cross correlations seems to be somewhat correlated with the foot angiosome concept.

**Table 3:** Percentages for the number of signs of lag time at both baseline and release periods in all subjects for repeat measurement 3 times, according to supine and 90° head-up tilt postures. Bold numbers stand for the highest value among three different situations for the lag time expression.

Posture	Sign of lag time	Ch.1 $\otimes$ Ch.2	Ch.1 $\otimes$ Ch.3	Ch.1 $\otimes$ Ch.4	Ch.2 $\otimes$ Ch.3	Ch.2 $\otimes$ Ch.4	Ch.3 $\otimes$ Ch.4
Supine	+	**60**	35	**65**	15	28.33	**66.67**
	0	23.33	26.67	18.33	26.67	**50**	20
	−	16.67	**38.33**	16.67	**58.33**	21.67	13.33
		100%	100%	100%	100%	100%	100%
Tilt	+	**65**	**51.67**	**71.67**	6	**51.67**	**70**
	0	23.33	31.67	18.33	35	35	23.33
	−	11.67	16.67	10	**58.33**	13.33	6
		100%	100%	100%	100%	100%	100%

### Power spectral density (PSD) Changes depending on reactive hyperemia

#### Representative results in one subject

**Figure 2:**
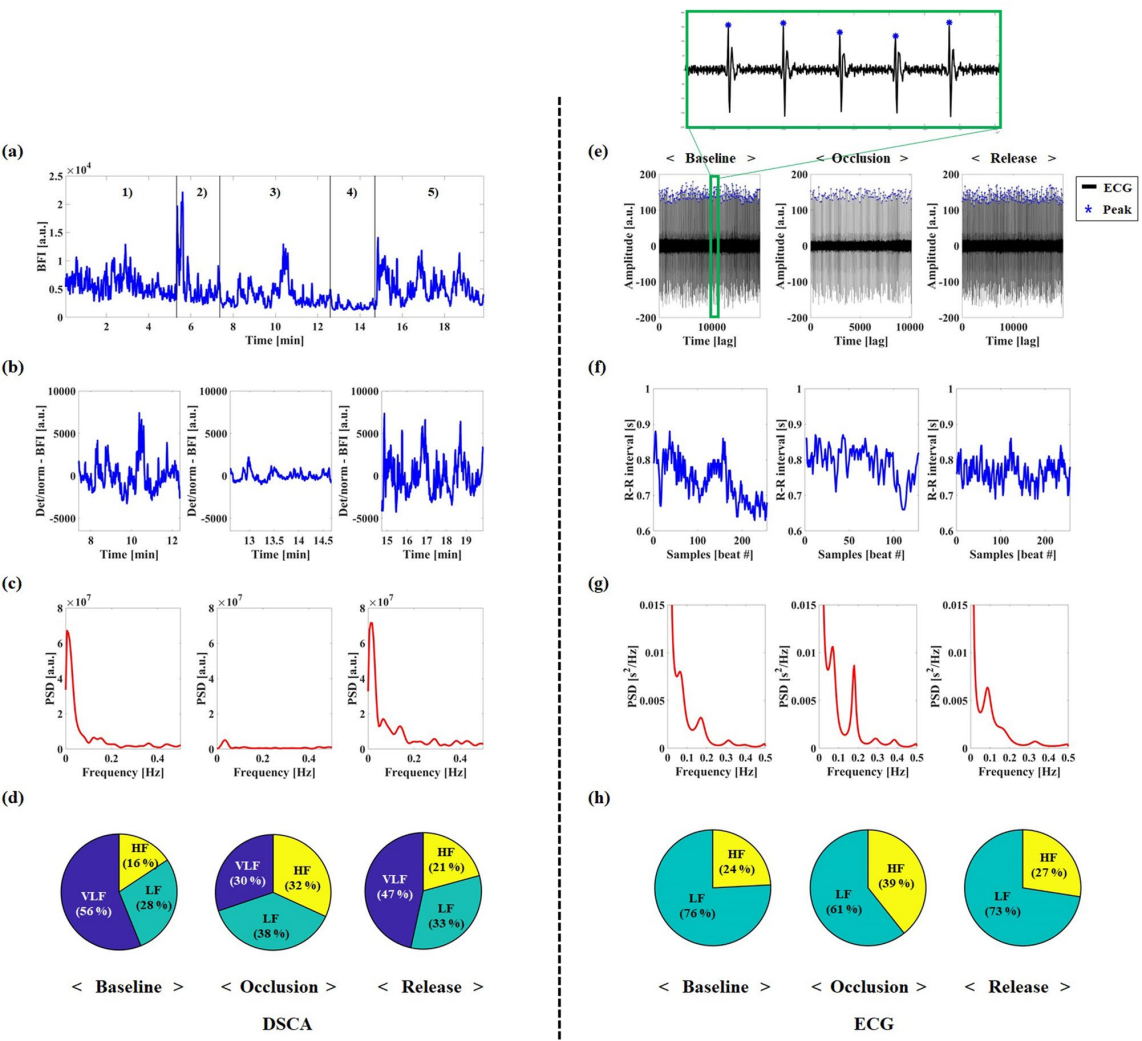
Representative results for PSD by DSCA (**a**–**d**) and ECG (**e**–**h**) in one subject, according to baseline, occlusion, and release periods during 90° head up tilt posture. (**a**) Raw BFIs in channel 4. (1) Baseline period in supine posture. (2) Posture change period. (3) Baseline period in 90° head-up tilt posture. (4) Cuff-occlusion period in 90° head-up tilt posture. (5) Release period in 90° head-up tilt posture. (**b**) Detrended and normalized processed BFI from sectioned BFI in channel 4. (**c**) PSD analysis in channel 4. (**d**) Relative PSD pie analysis among three frequency bands in channel 4. (**e**) Detrended and normalized ECG from sectioned ECG data. (**f**) HRV (R–R interval) from ECG signal. (**g**) PSD analysis from HRV. (**h**) Relative PSD pie analysis from HRV.

In order to analyze the PSD distribution changes for the VLF, LF, and HF ranges with respect to reactive hyperemia, we simultaneously measured the tissue perfusion and heart rate signals using DSCA and ECG, respectively. Figure 2 shows the representative results measured by DSCA (left side in Fig. 2) on the foot and by ECG (right side in Fig. 2) on the whole body from a single subject. In the DSCA measurements, we investigated the frequency oscillation changes of the VLFs (0.001–0.04 Hz), LFs (0.04–0.15 Hz), and HFs (0.15–0.4 Hz) rather than focusing on the change of the LFOs (0.01–0.15 Hz), in order to obtain the spectrum of VLF and to compare the PSD changes of LFs and HFs obtained by ECG. Firstly, we performed timeline selection and detrending/normalization without BPF to acquire entire oscillations during the periods of baseline (90° head-up tilt), occlusion, and release in raw BFI data from the channel 4. Then, we achieved the power spectral analysis with Welch’s method as a non-parametric approach in the frequency range from 0 to 0.5 Hz, as shown in Fig. 2(c). To estimate the changes in PSD distributions for the VLFs, LFs, and HFs with respect to reactive hyperemia, we analyzed the absolute PSD distribution. In the baseline period (90° head-up tilt), the absolute PSDs for the VLFs, LFs, and HFs were $2.20 \times 10^{6}$, $1.10 \times 10^{6}$, and $0.61 \times 10^{6}$ [a.u.], respectively. For the absolute PSDs of the VLFs, LFs, and HFs in the occlusion period, the values were $0.13 \times 10^{6}$, $0.16 \times 10^{6}$, and $0.14 \times 10^{6}$ [a.u.], respectively. In the occlusion period, the absolute PSD of VLFs dramatically decreased by about 17 times, and the absolute powers of LFs and HFs decreased by 7 and 4 times, respectively. In the release period, the absolute PSDs for the VLFs, LFs, and HFs were $2.36 \times 10^{6}$, $1.66 \times 10^{6}$, and $1.05 \times 10^{6}$ [a.u.], respectively, showing recovery to the baseline state via autoregulation. For the analysis of relative PSD distribution changes from DSCA, we examined a proportion of the absolute PSD for each frequency band within total frequency range (0–1.5 Hz) at the sampling rate of 3 Hz. At the baseline period, the relative PSD of VLFs, LFs, and HFs were 38.97, 19.56, and 10.88 [%], respectively. The relative PSD distributions at the occlusion period were 12.83 (VLF), 16.13 (LF), and 13.53 (HF) [%], respectively. In the relative PSD analysis, the proportion of VLFs and LFs decreased by about 26% and 3%, respectively, whereas that of HFs increased by 3%. At the release period, the relative PSD of VLFs, LFs, and HFs were 27.05, 18.97, and 12.06 [%], respectively. In order to examine the relative PSD distribution changes among three frequency bands for reactive hyperemia, based on the proportion of the absolute PSD, we performed pie chart analysis of the PSD distributions, as shown in Fig. 2(d). The relative PSD distribution of VLFs decreased by 26% in the occlusion period and recovered to the baseline values in the release period. In contrast to VLFs, the relative PSD distributions of the LFs and HFs increased by 10% (LFs) and 16% (HFs), respectively.

For the ECG results, we first performed timeline selection and detrending/normalization with the raw ECG data according to reactive hyperemia, as shown in Fig. 2(e). Next, we analyzed the HRV signals by estimating the variations in the heart rates and R–R intervals from peak detection in the raw ECG, as displayed in Fig. 2(f). Figure 2(g) shows the results of the power spectral analysis using an autoregressive model as a parametric approach from the HRV in the frequency domain. In contrast to the PSD distribution of the DSCA, two distinct peaks were seen in the LFs and HFs. In the baseline period, the absolute PSDs of the LFs and HFs were 738.28 and 235.64 [ms^2^], respectively. The absolute PSDs of the LFs and HFs were respectively 596.13 and 385.34 [ms^2^] in the occlusion period and 521.41 and 197.45 [ms^2^] in the release period. As shown in Fig. 2(h), we analyzed the relative PSD pie charts with LFs and HFs, except for the VLFs, because the absolute PSD distribution of the VLFs is relatively dominant among three frequency bands, and its origin has not been fully discovered in the ECG study. The changes in PSD distributions for the LFs showed 15% decrease between baseline and occlusion, and 12% increase between occlusion and recovery. For the PSD distribution of the HFs, this increased by 15% for the occlusion period.

#### Results of statistical analysis in all subjects

**Figure 3:**
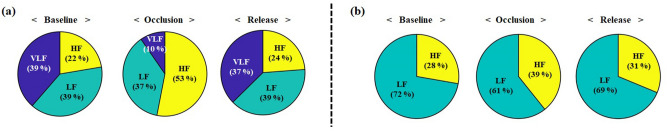
The pie charts of relative PSD distributions among frequency bands according to baseline, occlusion, and release periods in all subjects during 90° head up tilt posture. (**a**) DSCA measurement in channel 4. (**b**) ECG measurement.

As shown in Table 4, we performed statistical analyses with the PSD distributions for the three different frequency ranges, and the data were acquired by both DSCA from channel 4 and ECG with respect to reactive hyperemia. Table 4 shows average and standard deviation of relative PSD distributions from DSCA and ECG measurements. In the DSCA results, because of high standard deviations of the absolute PSD distributions between inter- and intra-subjects, we analyzed relative PSD distributions as the proportion of absolute power within total frequency range. The relative PSD of VLFs among three frequency bands showed the highest changes from baseline to release. The changes in the PSD distributions for the VLFs and LFs were related to magnitude changes in the raw BFIs owing to the effects of autoregulation of blood flow. In the ECG results of Table 4, the absolute PSD distribution of the LFs changed following cuff-occlusion and release, while the absolute power of the HFs did not show any significant differences. Comparing the two results by DSCA and ECG for the reactive hyperemia protocol, both slight decreases in LF band were correlated between the relative (DSCA)- and absolute (ECG) PSD distributions, while the distributions in HF band were not correlated. Figure 3 shows the relative PSD distribution changes among three frequency bands by DSCA and two frequency bands by ECG according to reactive hyperemia. These results were similar to the previous representative results (Fig. 2(d) and (h)) obtained from one subject. The relative PSD for VLFs and LFs decreased and increased from baseline to release in both modalities. The relative PSD distribution changes have similar tendencies between the VLFs and LFs obtained by DSCA and the LFs obtained by ECG. From the PSD distribution change analysis from DSCA measurements, it is worth noting that the PSD distribution for VLFs is remarkably reduced and increased following reactive hyperemia. Therefore, we can state that the frequency oscillation measurements by the DSCA system are especially sensitive to PSD distribution changes of the VLFs in specific tissues, which cannot be estimated from the ECG methodology.

**Table 4:** Average and standard deviation of relative (DSCA)- and absolute (ECG) PSD distributions for VLF, LF, and HF, according to baseline, occlusion, and release periods in all subjects during 90° head up tilt posture. Note that DSCA data was obtained from channel 4. *P < 0.05 compared to baseline period.

Freq	DSCA [%]			ECG [ms^2^]		
	Baseline	Occlusion	Release	Baseline	Occlusion	Release
VLF	38.59 ± 10.24	9.65 ± 7.15***	37.33 ± 9.40	–	–	–
LF	38.59 ± 7.60	37.28 ± 8.04	**38.87** ± 6.17	782.52 ± 230	523.00 ± 235	623.11 ± 106
HF	22.34 ± 8.26	53.07 ± 12.83**	23.80 ± 9.21	300.95 ± 170	309.71 ± 129	302.66 ± 167

#### PSD changes depending on 90° head-up tilt protocol

##### Representative results in one subject

**Figure 4:**
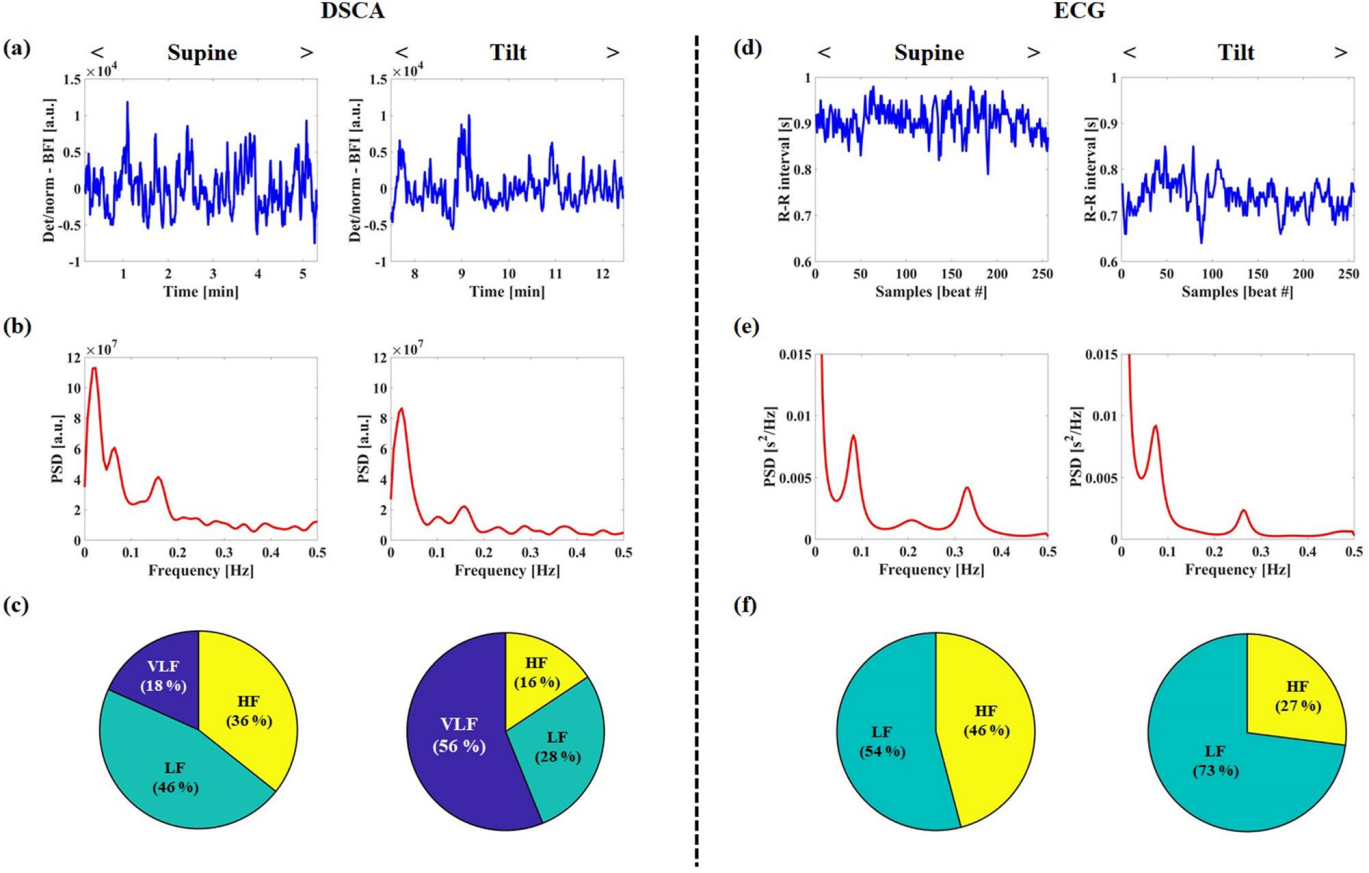
Representative results for PSD by DSCA (**a**–**c**) and ECG (**d**–**f**) in one subject, according to supine and head up tilt postures. (**a**) Detrended and normalized BFI from sectioned BFI in channel 4. (**b**) PSD analysis in channel 4. (**c**) PSD pie analysis among three frequency bands in channel 4. (**d**) HRV (R–R interval) from ECG signal. (**e**) Relative PSD analysis from HRV. (**f**) PSD pie analysis from HRV.

Several years ago, Malik et al.^13^ reported that the relative PSD distribution of LFs measured by ECG exceeded that of HFs at the 90° head-up tilt, compared to the supine posture. In order to estimate the PSD distribution change of entire frequency oscillations (VLF, LF and HF) achieved by DSCA according to the head-up tilt protocol, we simultaneously performed the PSD analysis with the HRV signal, as shown in Fig. 4. In the DSCA results of Fig. 4(a–b), the peaks in the VLFs (0.001–0.04 Hz), LFs (0.04–0.15 Hz), and HFs (0.15–0.4 Hz) ranges were changed between the two postures. In the supine state, the absolute PSD distributions for the VLFs, LFs, and HFs in channel 4 were $0.61 \times 10^{6}$, $1.54 \times 10^{6}$, and $1.20 \times 10^{6}$ [a.u.], respectively. In the tilt posture, the absolute PSD distributions of the three spectra were $2.20 \times 10^{6}$ (VLF), $1.10 \times 10^{6}$ (LF), and $0.61 \times 10^{6}$ (HF) [a.u.]. The relative PSD distributions of VLFs, LFs, and HFs at the supine posture, for the proportion of the absolute PSD within total frequency range (0–1.5 Hz), were 8.51, 21.41, and 16.61 [%], respectively. At the tilt posture, the relative PSD distributions for VLFs, LFs, and HFs were 38.97, 19.56, and 10.88 [%], respectively. As shown in Fig. 4(c), for the pie chart analysis of the relative PSD distributions among three frequency bands, the VLF distribution were changed in the tilt posture (VLF: 38% increase; LF: 18% decrease; HF: 20% decrease) compared to that of the supine state.

In the ECG results (Fig. 4(d–f)), we observed similar tendencies for the PSD distribution changes of LFs and HFs as those in Malik et al.^13^. For the absolute PSD distributions, the LFs and HFs were respectively 430.83 and 365.63 [ms^2^] at the supine posture and 422.79 and 156.45 [ms^2^] at the tilt posture. In the PSD pie analysis from HRV (Fig. 4(f)), the relative distribution of the LFs increased by 19% according to the posture change.

##### Results of statistical analysis in all subjects

**Figure 5:**
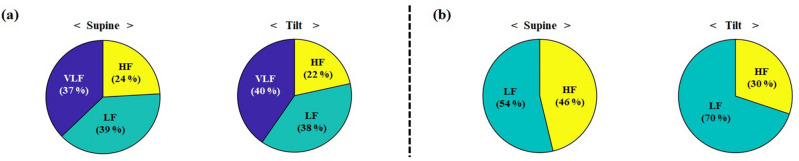
The pie charts of relative PSD distributions among frequency bands according to baseline, occlusion, and release periods in all subjects during 90° head up tilt protocol. (**a**) DSCA measurement in channel 4. (**b**) ECG measurement.

Table 5 shows the statistical analyses of the relative (DSCA)- and absolute (ECG) PSD distributions for the three different frequency ranges, with respect to the supine and head-up tilt postures. In DSCA, we analyzed the proportions of the absolute PSD within total frequency range, which is the same procedure in Table 4. The relative PSDs for both of VLFs and LFs at the tilt posture were higher than that of the supine posture, and the relative proportion of VLFs showed the highest change among the three frequency bands. However, the relative PSDs for the HFs were nearly unchanged. In the ECG results, the highest change in absolute power was noted for the PSD distribution of HFs. Figure 5 shows the pie charts for the relative PSD distributions among three frequency bands (DSCA) and two frequency bands (ECG). From the DSCA result of Fig. 5(a), we noted that the relative PSD distribution of HFs decreased by 2%. From the ECG result of Fig. 5(b), the PSD distribution of the HFs decreased significantly. Although the change of the PSD distribution of the HFs in DSCA measurement was little, a decreasing tendency was found from the both results.

**Table 5:** Average and standard deviation of relative (DSCA)- and absolute PSD distributions for VLF, LF, and HF, according to supine and head up tilt postures in all subjects. Note that DSCA data were obtained from channel 4. * *P* < 0.05 compared to supine posture.

Freq	DSCA [%]		ECG [ms^2^]	
	Supine	Tilt	Supine	Tilt
VLF	20.61 ± 8.92	25.05 ± 9.26**	–	–
LF	21.48 ± 5.52	23.59 ± 6.11*	522.00 ± 123.82	576.37 ± 242.43
HF	13.53 ± 3.14	13.37 ± 3.22	482.36 ± 234.47	250.76 ± 181.27*

## Discussions

In this study, we explored cross-correlation analysis with LFOs obtained using the DSCA system based on the foot angiosome concept ^5^. From the cross-correlation analysis, it is observed that cross correlations do not strongly support the foot angiosome concept in terms of the micro-perfusion oscillations. As shown in Table 2, by analyzing the cross-correlation amplitudes among the four optical probes, we found that the only relationship between two probes in the LPB angiosome followed a previous hypothesis. For the angiosome concept to be valid, the Ch.1 $\otimes$ Ch.3 and Ch.2 $\otimes$ Ch.4 pairs should both have high maximum values among the six relationships because tissue perfusions in the same angiosome must share the same artery. Only the Ch.2 $\otimes$ Ch.4 pair located in the LPB angiosome space was compatible with our hypothesis. For slight inconsistencies in the average ranks for maximum values of the cross correlations with respect to the baseline/release periods and tilt/supine postures, one possible explanation is that reactive hyperemia and posture change may alter the origins of the LFOs, such as the myogenic or metabolic theory of autoregulation^26^.

As shown in Table 3, in the lag time analysis of the cross correlations, we found two reproducible results. The first finding was that the LFOs in both channels 1 and 3 in the MPB angiosome space were faster than those in channels 2 and 4 in the LPB angiosome, which is compatible with arterial paths in the sole of the foot, as expressed by the red lines in Fig. 1(a). Since the PTA branches out from the heel of the foot to the MPB and LPB, and the MPB is closer to the CB of the PTA, the blood supplies to areas monitored by channels 1 and 3 involved in the MPB angiosome arrive earlier than those of the LPB angiosome (areas monitored by channels 2 and 4). The second finding was that in the relationships between both channels in the same angiosome (i.e., Ch.1 $\otimes$ Ch.3 and Ch.2 $\otimes$ Ch.4), high percentages for the lag time signs were ambiguous with respect to the supine/tilt postures. Although this is not compatible with physiological arterial blood flow, there may be two possible explanations: (1) In the same angiosome space, the blood flow may be fast enough that it is hard to distinguish the time latency between two channels within the sampling speed resolution. (2) The plausibility of the foot angiosome concept being incorrect may be attributable to the circulatory anatomies^27,28^. As shown in Fig. 1(a), the arteries in the regions of channels 1 and 2 meet each other between the LPB and MPB angiosome spaces at the top of the foot. Since the arteries that form the two different angiosomes are linked, the arterio-arterial anastomosis eventually renders their boundary blurry so that the accuracy of the lag latency analysis in the same angiosome may decrease. Depending on the supine and tilt postures, although the lag time analysis in the same angiosome seems unclear, the evident time difference in the hemodynamic responses between the different angiosome spaces seems to support the foot angiosome concept at the microcirculation level. Note that the validity of the angiosome concept is therefore still controversial^10–12^.

Based on Table 1, considering another correlation analysis, we quantified six Pearson’s correlation coefficients which can estimate temporal similarity of the pairs. Table 6 shows the ranks between six cross correlations and six Pearson’s correlation coefficients in a single subject, when the lag time is zero. Both ranks by cross-correlation and Pearson’s correlation coefficients had similar tendencies at the baseline and release periods, although they were not exactly the same each other. This is because the coefficients of the cross-correlation analysis have been normalized. Compared to those of the baseline and release periods, the six Pearson’s correlation coefficients at the occlusion period showed lower values close to zero, resulting in − 0.0695 (Ch.1 $\otimes$ Ch.2), -0.0105 (Ch.1 $\otimes$ Ch.3), 0.2398 (Ch.1 $\otimes$ Ch.4), 0.3152 (Ch.2 $\otimes$ Ch.3), 0.3140 (Ch.2 $\otimes$ Ch.4), 0.4747 (Ch.3 $\otimes$ Ch.4). As shown in Table 7, from statistical analysis (N = 10) for the ranks of six Pearson’s correlation coefficients, we observed that high ranks were still in three-relationships (Ch.2 $\otimes$ Ch.3, Ch.2 $\otimes$ Ch.4, and Ch.3 $\otimes$ Ch.4), which was also found in the high ranks from the six cross correlations. This implies that the analysis of Pearson’s correlation coefficient also does not fully support to the foot angiosome concept. To date, regarding the angiosome concept, some studies have focused on assessment of peripheral tissue perfusion to identify the origin location of diabetic foot ulcers with bench-top optical modalities^29–34^, to evaluate the quantitative wound healing with high-resolution imaging modalities such as CT^35,36^, SPECT/CT^37,38^ and MRI perfusion^39–41^, and to evaluate leg revascularization surgeries in CLI patients^11^. To the best of our knowledge, no studies have reported the assessment of cross correlation relationships between the inter- and intra-tissue spaces based on the angiosome concept. The main objective of this study was to explore the foot angiosome concept from the view of the microcirculation level by a novel approach compared to the previous studies. Therefore, we expect that our results, which partially support the angiosome concept, might help to consolidate the foot angiosome in tissue perfusion, with an understanding of the physiological background.

**Table 6:** Comparison of ranks between six cross-correlations and six Pearson’s correlation (CORR) coefficients at zero lag and in supine posture according to baseline and release periods from one subject’s foot.

Periods	Contents	Ch.1 $\otimes$ Ch.2	Ch.1 $\otimes$ Ch.3	Ch.1 $\otimes$ Ch.4	Ch.2 $\otimes$ Ch.3	Ch.2 $\otimes$ Ch.4	Ch.3 $\otimes$ Ch.4
Baseline	Max. value [a.u.]	$4.28 \times 10^{6}$	$2.17 \times 10^{6}$	$2.52 \times 10^{6}$	$19.02 \times 10^{6}$	$36.05 \times 10^{6}$	$11.26 \times 10^{6}$
	Rank	4	6	5	2	1	3
	Lag time [s]	0	0	0	0	0	0
	Pearson’s CORR coefficient (Rank)	0.3398 (5)	0.4323 (4)	0.2906 (6)	0.7826 (2)	0.8542 (1)	0.6720 (3)
Release	Max. value [a.u.]	$8.02 \times 10^{6}$	$4.03 \times 10^{6}$	$5.22 \times 10^{6}$	$16.18 \times 10^{6}$	$25.86 \times 10^{6}$	$21.18 \times 10^{6}$
	Rank	4	6	5	3	1	2
	Lag time [s]	0	0	0	0	0	0
	Pearson’s CORR coefficient (Rank)	0.6746 (3)	0.5485 (5)	0.3580 (6)	0.7274 (2)	0.5920 (4)	0.7883 (1)

**Table 7:** Average and standard deviation of the rank for Pearson’s correlation coefficients at baseline and release periods in all subjects, according to supine and 90° head-up tilt postures.

Posture	Periods	Ch.1 − Ch.2	Ch.1 − Ch.3	Ch.1 − Ch.4	Ch.2 − Ch.3	Ch.2 − Ch.4	Ch.3 − Ch.4
Supine	Baseline	4.30 ± 0.94	4.03 ± 0.84	4.37 ± 0.66	3.10 ± 1.18	2.30 ± 0.90	2.90 ± 0.57
	Release	3.63 ± 0.74	4.03 ± 0.74	4.30 ± 0.96	3.13 ± 1.32	2.37 ± 0.81	3.35 ± 0.72
Tilt	Baseline	4.10 ± 0.74	4.06 ± 1.15	4.33 ± 0.59	3.20 ± 0.89	2.50 ± 0.96	2.80 ± 0.97
	Release	3.83 ± 1.02	3.83 ± 0.71	4.63 ± 0.78	2.70 ± 0.94	2.90 ± 1.07	3.10 ± 1.03

From the PSD change analyses using DSCA, we confirmed that the DSCA system could effectively distinguish tissue perfusion and its fluctuations on the sole of the foot following reactive hyperemia and the 90° head-up tilt protocol, as effects of the autoregulation of blood flow and spontaneous systemic changes. Although we included the results for PSD distribution analyses with only channel 4 data, the results for all the other channels showed similar trends, and the sensitivity in channel 4 was the best among the four channels. As shown in Table 4 and Fig. 3, in the reactive hyperemia experiment, the remarkable reduction in PSD distribution of the VLFs among the three frequency bands at the cuff-occlusion period implies that the DSCA system probes the vasomotion tone inside the tissues in the VLF band and not the noise information. As shown in Table 4, we observed that the PSD distribution of HFs in the DSCA measurements following the reactive hyperemia were relatively increased because of dominant reduction of the relative PSD in VLF band. As shown in Table 5 and Fig. 5, however, the PSD distributions of VLFs and LFs measured by DSCA in the 90° head-up tilt protocol experiment, increased at the tilt posture and that of VLF band was mostly changed. In Table 5, the relative PSDs of HFs by DSCA did not change significantly from supine to head up tilt posture. As reported in Tong et al.^17^, we also experienced that all subjects had no clear peaks at the HF band which are closely related with the respiratory frequency. Moreover, it was limited to scope the HF band signal with the relatively low sampling rate of DSCA at the regional tissue.

In Table 4 and Fig. 3, for the analysis of PSD changes by ECG, in the reactive hyperemia protocol, we found that the LF distribution in the occlusion period somewhat decreased compared to the HF distribution for the baseline, thus showing that the ratio of LFs to HFs before and after occlusion were 2.57 and 1.56, respectively. By occluding the femoral artery in the leg, the reactive hyperemia protocol mimics a situation of peripheral arterial disease (PAD), which is a circulatory problem associated with narrowed arteries and reduced blood supply to the limbs. After cuff occlusion, the decrease in PSD distribution of the LFs was similar to a previous report that the LF distribution was lower in patients with cardiovascular morbidities with PAD than patients without PAD, as observed by Chen et al.^42^. As shown in Fig. 4(d–f), in the 90° head-up tilt protocol, the change in LF distribution by ECG was similar to that observed by Malik et al.^13^, where the absolute power of the LFs slightly decreased at tilt posture whilst the relative distribution of LFs considerably increased. From simultaneous measurements using DSCA and ECG, it was observed that there was a relationship between the LF distribution obtained from HRV analysis of ECG and VLF and LF ($\approx$ LFO) distributions obtained using DSCA.

The reason why the results of the PSD analyses estimated using DSCA and ECG were not similar was attributable to the measurement scope of each system being different. The ECG measurements are used to estimate all systemic oscillations in the human body. In contrast, the DSCA estimates the relative blood flow and perfusion oscillations in specific tissues. Although the origin of the VLFs has not yet been fully realized from ECG studies, the basis by which we obtained the VLFs (0.001–0.04 Hz) and LFs (0.04–0.15 Hz) using DSCA might be affected by endothelium-related metabolic (0.008–0.02 Hz), neurogenic (0.02–0.05 Hz), and myogenic (0.05–0.15 Hz) regulations in skin blood flow oscillations^43^. In this work, the DSCA probed the LFOs at the sole of the foot. Therefore, we suggest that the ECG is useful for diagnosis of various diseases, such as cardiovascular diseases, diabetes, and so on, but the DSCA system has broad impact for diagnosing localized diseases (e.g., CLI, PAD, cancers, diabetic foot ulcer) especially at the tissue level, for which specific diseases cannot be diagnosed by ECG.

Thus far, to our knowledge, there are only a few reported studies on tissue perfusion fluctuation analysis with optical systems, which can measure hemodynamics, that focus on analyzing the relative changes or differences in blood oxygenation, volume, and flow between healthy subjects and patients. If the previous measurement parameters and LFOs (or VLFs; LFs) are together applied to patients, we expect to be able to scope these diseases more precisely and gain more resources in clinical situations. To this end, we are planning to implement a high-speed diffuse optical system that can estimate the tissue metabolic rate of oxygen consumption for scoping a broader power spectrum within the tissue.

## Conclusions

We performed a pilot study for validation of the foot angiosome concept with a correlation analysis of the LFOs obtained using the DSCA system; our results in this study do not strongly support the angiosome concept. However, because the angiosome concept is gradually gaining more attention in terms of treatments for patients with CLI, our results can help improve insights on tissue perfusion assessments. As shown in this work, we verified that the DSCA can estimate VLF and LF ($\approx$ LFO) changes in specific tissues during arterial cuff-occlusion and 90° head-up tilt protocols. In future work, we intend to compare the results between diabetic patients and healthy subjects to assess the key factors distinguishing these two groups, so that early diagnosis of diabetic complications may be possible. To date, some research groups have studied the early diagnosis of diabetic complications using optical systems with near-infrared sources^44–46^. However, these studies have focused on the time-averaged hemodynamic variables and disregarded LFO information. Since tissue perfusion and its oscillations are important biomarkers in human vascular health, it is expected that these factors will offer new insights into diagnosis and therapeutic monitoring of diabetic complications.

## Materials and methods

### Participants

The present experimental protocol was approved by the Institutional Review Board (IRB) of Daegu Gyeongbuk Institute of Science and Technology (approval no. DGIST-190829-h-072-01). Ten healthy subjects (mean age: 26.5 years old; 7 males and 3 females) participated in this study on tissue perfusion measurements on the foot. Prior to and during the experiments, we performed in accordance with relevant guidelines/regulations for the approved research and obtained the informed consent for the experimental protocol from all participants, and they were asked to avoid smoking and drinking caffeinated and alcoholic beverages for minimizing the vasoactive effects and to limit movements as much as possible for minimizing motion artifacts in the DSCA and ECG signals.

### Experimental situation and protocol

**Figure 6:**
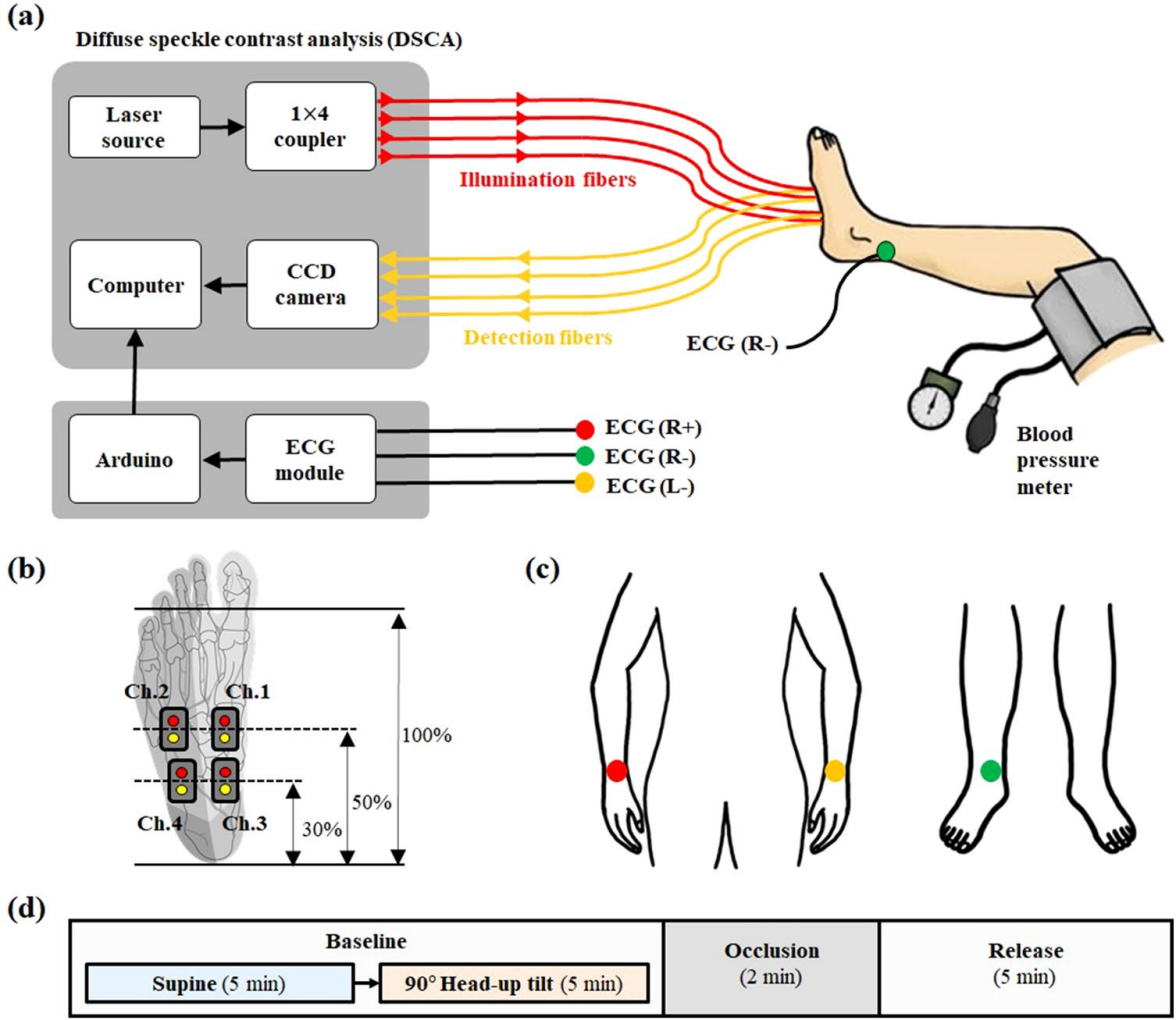
Experimental situation using DSCA and ECG. (**a**) Schematic diagram of the tissue perfusion measurement with DSCA and heart rate variability measurement with ECG. (**b**) The criteria of DSCA measurement positions in subject’s sole of foot. Red and yellow circles stand for illumination and detector fibers, respectively. (**c**) ECG measurement positions. (**d**) Flow chart of the measurement protocol.

Figure 6(a) shows the experimental schematic for the hemodynamic response measurements from the sole of the foot using DSCA and via HRV measurements from the human body using ECG. The subjects’ thighs were wrapped using a blood-pressure cuff to temporarily block the blood flow from the femoral artery to the foot. For simultaneous measurement of tissue perfusion and HRV, the four-channel optical probes of the DSCA system were attached to the right foot, and the three-channel ECG probes were attached to left and right wrists and right ankle, as shown in Fig. 6b and c. The reason behind the simultaneous measurements using DSCA and ECG was to evaluate the relevance between the frequency oscillations within specific tissues and systemic oscillations in the entire body.

For the ECG record, three ECG sensor patches were linked to an ECG sensor module (AD8232, Heart Rate Monitor, EDUINO). The ECG signal obtained by the ECG module was sent to a computer using an Arduino Uno module. In the DSCA configuration, a laser diode (DL-785-100S, CrystaLaser, 785 nm, 100 mW) was linked to a 1 × 4 coupler with multi-mode optical fibers to extend the system to four channels. To reduce the harmful effects from large light power illumination, all lasers at the ends of the illumination optical fibers were adjusted to apply around 6.5 mW^47^. The scattered light was detected using a CCD camera (F-033B, Stringray) through four single-mode optical fibers. The optical probes (pairs of laser source and detector fibers) were attached on the foot through flexible polydimethylsiloxane (PDMS) for optical guidance. All of the inter-optode distances for the probes were set to 10 mm, so that the penetration depth was approximately 5 mm. More details on the probe configuration can be found in our previous paper^23^.

As shown in Fig. 6(b), all the measurement positions in each subject’s foot were determined by the individual foot sizes. Channels 1 and 3 (Ch.1 and Ch.3) were located on areas vascularized by the MPB and channels 2 and 4 (Ch.2 and Ch.4) were located on areas vascularized by the LPB to investigate possible correlations between LFOs from different positions on the foot angiosome. We hypothesized that the optical probe pairs (Ch.1 $\otimes$ Ch.3 and Ch.2 $\otimes$ Ch.4) located at the same angiosome will have higher correlations than those from different ones (Ch.1 $\otimes$ Ch.4, Ch.1 $\otimes$ Ch.2, Ch.2 $\otimes$ Ch.3, and Ch.3 $\otimes$ Ch.4) since the microcirculation in any specific angiosome will share the same origin of LFOs.

In the experimental protocol, three periods were defined as baseline, cuff-occlusion, and release of occlusion, as shown in Fig. 6(d). In the baseline period, to analyze the PSD changes of VLFs, LFs, and HFs according to the head-up tilt protocol^13^, the measurements involve a supine posture for 5 min and 90° head-up posture for 5 min. Between the two postures, there was a 2 min measurement interval that was removed from analysis as a waiting period for the settling time to achieve stable vasomotion because dramatic posture changes can affect vascular tone. In the occlusion period, a cuff-occlusion pressure of about 200 mmHg was induced for 2 min to change the physiological blood flow to the leg and to verify that the DSCA instrument could detect hemodynamic changes according to reactive hyperemia. The release period is the post-occlusion of the pressure for 5 min. Therefore, the measurement time for each subject was about 19 min. For better statistical analysis, these measurement procedures were repeated six times with gaps of 2 h rest for each subject. In each measurement phase, the sequence of supine and head-up tilt postures was changed for a more accurate analysis of the PSD changes.

### Data processing

In the ECG record, the signal was acquired via serial communication using an Arduino Uno with 9600 board rates, such that the data acquisition speed was 100 Hz. In the DSCA system, the image acquisition from the CCD camera was achieved via the LabVIEW program with an exposure time of 10 ms and sampling rate of 60 fps. The BFI within a volume of tissue was calculated using the speckle intensity on the region of interest (ROI). For detailed theoretical descriptions of the DSCA, we refer the readers to published literature^20^. In signal processing for speckle contrast calculation, there are two types of analysis for the temporal and spatial domains. For the temporal domain analysis, the speckle contrast can be obtained from the correlation of a group of pixels over a fixed position and at successively different times. In contrast, for the spatial domain analysis, the speckle contrast can be obtained from statistics over n × n pixels out of the entire image at a fixed time. In this work, we utilized the temporal domain analysis to simultaneously acquire multi-channel data with a 9 × 9 pixels window as the ROI. The temporal speckle contrast ($K_{t}$) is defined as in Eq. (1).

1 $$K_{t} = {\raise0.7ex\hbox{${\sigma_{t} }$} \!\mathord{\left/ {\vphantom {{\sigma_{t} } {\left\langle I \right\rangle }}}\right.\kern-\nulldelimiterspace} \!\lower0.7ex\hbox{${\left\langle I \right\rangle }$}}$$

where $\sigma_{t}$ and $\left\langle I \right\rangle$ are the temporal standard deviation and average of light intensities over 20 frames, respectively. Therefore, the data acquisition speed was 3 Hz when using 20 images at 60 fps. It is possible to increase the sampling rate above 3 Hz using a high-speed camera, but the camera used in this study was limited to 60 fps because of the maximum frame rate of the CCD camera. In addition, we did increase the sampling rate above 3 Hz because the LFOs did not exceed 0.15 Hz. Finally, we defined $1/K_{t}^{2}$ as the BFI with arbitrary unit, which varies linearly with physiological blood flow.

### Data analysis

#### Cross-correlation analysis from DSCA signals

**Figure 7:**
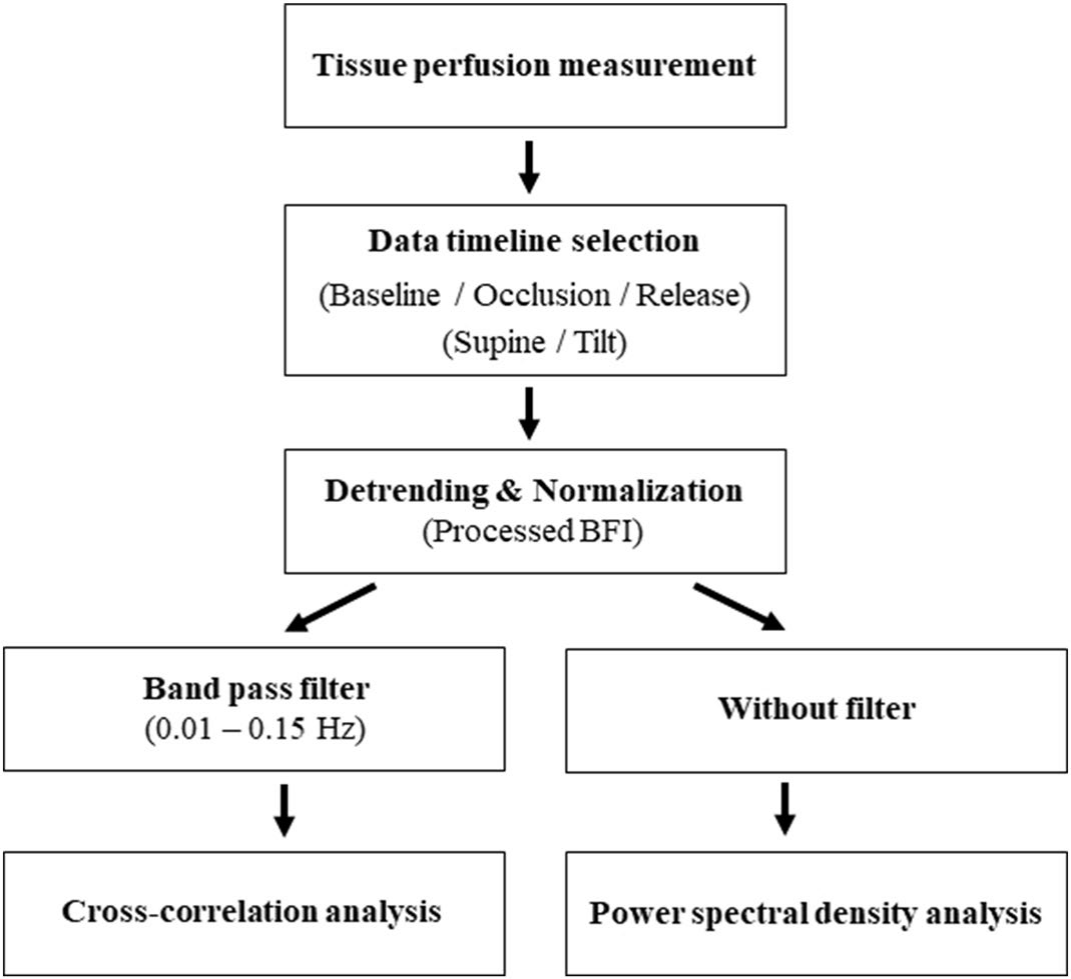
Flow chart of data analysis for cross-correlation analysis and power spectral analysis in DSCA.

After tissue perfusion measurements on the foot with the DSCA system, we analyzed the cross-correlation relationships among the four channels, as shown in Fig. 7. Within the time-series tissue perfusion raw data, we chose the timeline selections according to the baseline, occlusion, and release periods in the supine and head-up tilt postures. The reason for selections of the occlusion and release periods was to ascertain that the cross-correlation relationship still remained among the four probes after baseline period. Before applying the cross-correlation analysis, the selected matrices (4 channels × n-time-series: 4 × n matrix) were processed by detrending and normalization to remove linear trends and obtain better cross-correlation analysis. Next, the detrended and normalized data are transferred into a band pass filter with the pass band frequencies from 0.01 Hz to 0.15 Hz to deal with the LFOs range. Finally, we employed an unbiased cross correlation procedure using MATLAB software, for the cross-correlational analysis.

#### Heart rate variability (HRV) analysis from electrocardiographic signal

The HRV, which is obtained from the ECG record, represents one of the promising biomarkers to express oscillations in consecutive cardiac cycles^13^. Among several available measures of the HRV, we analyzed the variations of the instantaneous heart rate and R–R intervals. We obtained 256 consecutive R–R values in the baseline and release periods for the HRV analysis in the frequency domain. In the occlusion period, 128 consecutive R–R intervals were utilized because of limited measurement time (2 min).

#### Power spectral density (PSD) analysis from processed blood flow index (BFI) and HRV data

In both the processed BFI (detrended and normalized) and HRV data, the timeline according to the baseline, occlusion, and release periods in supine and head-up tilt postures were selected and processed by detrending and normalization. In order to acquire the entire oscillations in human body, we did not apply the filters such as low/high/band pass filters in both the BFI and HRV data. The time-series signals for the processed BFI and HRV data were converted into frequency domain signals. Next, spectral analysis was performed using (1) Welch’s method (triangular window; 900 samples) in a non-parametric approach^13^ for the processed BFI and (2) the autoregressive model (order 20) in a parametric approach^13^ for the HRV data in MATLAB software. Since the hemodynamic system in the sole tissue has not been modeled yet, the PSD analysis from DSCA was achieved by the non-parametric method. Then, both signals by DSCA and ECG were analyzed in the three frequency bands, such as VLFs (0.001–0.04 Hz), LFs (0.04–0.15 Hz), and HFs (0.15–0.4 Hz). In the case of HRV data, a pie chart analysis was done with the absolute power of the two frequency bands, to confirm the relative distributions between two frequency bands, LFs and HFs. In the processed BFI, we examined the relative PSD distributions in the three frequency bands from 0 to 1.5 Hz (sampling rate of DSCA: 3 Hz), due to the high variances of absolute PSD distributions from inter- and intra-subjects.

#### Statistical analysis

For statistical analysis, the averages and standard deviations of the ranks of the maximum values of the cross correlations and the absolute PSD distributions of the VLFs, LFs, and HFs were obtained with ten samples for the number of subjects (n = 10) and six-repeated measurement samples. A two-tailed paired t-test was performed with the significance level as a *p*-value < 0.05.
